# Wie stark trifft die Corona-Pandemie die Kliniken für Orthopädie und Unfallchirurgie? Eine Analyse der ersten 5 Wochen

**DOI:** 10.1007/s00132-020-03926-4

**Published:** 2020-05-20

**Authors:** Nikolaus von Dercks, Christian Körner, Christoph-E. Heyde, Jan Theopold

**Affiliations:** 1 Stabstelle Medizincontrolling, Universitätsklinikum Leipzig AöR, Liebigstraße 18, Haus B, 04103 Leipzig, Deutschland; 2 Bereich Finanzen und Controlling, Universitätsklinikum Leipzig AöR, Leipzig, Deutschland; 3 Klinik für Orthopädie, Unfallchirurgie und Plastische Chirurgie, Universitätsklinikum Leipzig AöR, Leipzig, Deutschland

**Keywords:** DRG, COVID-19-Krankenhausentlastungsgesetz, Orthopädie und Unfallchirurgie, Kompensationsmechanismen, Corona-Pandemie, Diagnosis-related groups, COVID-19 Hospital Relief Act, Orthopaedic and trauma surgery, Compensation mechanisms, Coronavirus pandemic

## Abstract

**Hintergrund:**

Die Corona-Pandemie stellt Krankenhäuser vor enorme finanzielle Herausforderungen. Am Beispiel einer Klinik für Orthopädie und Unfallchirurgie soll die Leistungsentwicklung in der stationären Versorgung der ersten 5 Wochen nach Beginn der gesetzgeberisch angeordneten Leistungsreduktion im Vorjahresvergleich sowie eine Bewertung der gesetzgeberisch festgelegten Kompensationsmaßnahmen bewertet werden.

**Methodik:**

Anhand der Leistungszahlen wird ein Vergleich des Zeitraumes 16.03. bis 17.04.2019 und demselben Zeitraum 2020 durchgeführt. Veränderungen von Fallzahl, Casemix, Casemix-Index und Daymix-Index sowie den Belegungstagen werden erfasst. Auf diese Veränderungen werden die monetären Maßnahmen aus dem COVID-19-Krankenhausentlastungsgesetz angewendet und deren Auskömmlichkeit bewertet.

**Ergebnisse:**

Im Vergleich zum Vorjahr kommt es im Beobachtungszeitraum zu einem Rückgang der stationären Aufnahmen von 307 Patienten. Demzufolge waren ein Rückgang des Casemix um 595 Punkte und der Belegungstage um 2320 Tage zu verzeichnen. Es ergibt sich ein Erlösrückgang von ca. 1,9 Mio. EUR. Die Leerbettenpauschale stellt die monetär größte Kompensation der Erlösausfälle dar. Sie beläuft sich auf ca. 1,3 Mio. EUR. Unter Berücksichtigung weiterer Unterstützung und einer Bereinigung um variable Kosten bleibt ein Fehlbetrag von 382.069 € in Bezug auf die stationären Leistungen für 5 Wochen.

**Diskussion:**

Die Maßnahmen des Gesetzgebers stellen eine wichtige Stütze zur wirtschaftlichen Absicherung deutscher Krankenhäuser dar. Die fehlende Differenzierung der Maßnahmen nach Fachrichtung führt für Orthopädie und Unfallchirurgie zu einer nur unzureichenden Kompensation.

## Abstract (en)

**Background:**

The corona pandemic poses enormous financial challenges for hospitals. Using the example of a clinic for orthopaedics and trauma surgery, the performance development of the first 5 weeks in comparison to last year and an evaluation of the compensation measures laid down by law will be evaluated.

**Methodology:**

Based on the performance figures, a comparison is made between the period 16.03 to 17.04.2019 and the same period in 2020. Changes in the number of cases, case mix, case-mix index and day-mix index, as well as the occupancy days are recorded. The monetary measures from the COVID-19 Hospital Relief Act are applied to these changes, and the adequacy is evaluated.

**Results:**

Compared to last year, there was a decrease in inpatient admissions of *n* = 307 patients in the observation period. As a result, there was a decrease of 595 points in case mix and 2320 days of occupancy. This results in a decrease in revenue of approximately EUR 1.9 million. The flat rate for empty beds represents the largest monetary compensation for the loss of revenue. It amounts to approximately EUR 1.3 million. Taking into account further support and an adjustment for variable costs, a deficit of EUR 382,069 remains for 5 weeks.

**Discussion:**

The measures taken by the Government are an important pillar for the economic security of German hospitals. The lack of differentiation of measures by specialty leads to insufficient compensation for orthopaedics and trauma surgery.

## Einleitung

Im Rahmen der Corona-Pandemie steht das deutsche Gesundheitssystem vor immensen Herausforderungen [8, 9, 11]. Hierzu zählt nicht zuletzt auch die wirtschaftliche Absicherung der Leistungserbringer. Vor diesem Hintergrund hat die Bundesregierung im März 2020 das „Gesetz zum Ausgleich COVID-19 bedingter finanzieller Belastungen der Krankenhäuser und weiterer Gesundheitseinrichtungen“ (COVID-19-Krankenhausentlastungsgesetz) beschlossen [4]. Darin enthaltene finanzielle Absicherungsmechanismen adressieren sowohl stationäre wie auch ambulante Leistungserbringer. Krankenhäuser werden u. a. dadurch berücksichtigt, dass der Pflegeentgeltwert vorübergehend von 146,55 € auf 185 € angehoben wird, der Aufbau von zusätzlichen Intensivbetten gefördert wird und ein finanzieller Ausgleich für nicht belegte Betten gezahlt werden soll [4]. Gerade der letzte Aspekt soll auch den Umstand berücksichtigen, dass planbare Operationen und andere Behandlungen verschoben werden müssen, um Infektionsrisiken zu vermeiden und Kapazitäten in den Krankenhäusern, insbesondere Intensivbetten, für infizierte Patienten zu schaffen [1, 8, 12]. Kliniken mit einem hohen Anteil an elektiven Eingriffen sind demnach von den Maßnahmen besonders betroffen und auf die finanziellen Entlastungsmaßnahmen angewiesen. Für voll- und teilstationär aufgenommene Patienten ab dem 1. April 2020 bekommt das Krankenhaus eine zusätzliche Vergütung von 50 € als Abgeltung für Preis- und Mengensteigerungen durch die Pandemie, insbesondere bei Schutzausrüstung [3, 6].

Ziel dieser Arbeit ist es, am Beispiel der Klinik für Orthopädie, Unfallchirurgie und Plastische Chirurgie eines Maximalversorgers, nach den ersten 5 Wochen der restriktiven Maßnahmen im Rahmen der Corona-Pandemie die Leistungszahlen für stationäre Behandlungen im Vorjahresvergleich zu betrachten. Darüber hinaus soll eine Abschätzung zur Auskömmlichkeit der finanziellen Ausgleiche getroffen werden.

## Studiendesign und Untersuchungs﻿metho﻿den

An der Klinik für Orthopädie, Unfallchirurgie und Plastische Chirurgie (KOUP) werden pro Jahr über 6000 Patienten stationär behandelt. Diese verteilen sich auf die 5 Bereiche Unfallchirurgie (UCh), Wirbelsäulenchirurgie (WCh), Arthroskopie/gelenkerhaltende Chirurgie (ArCh), Plastische Chirurgie (PCh) und Orthopädie (ORT). Für den Vergleich des Zeitraumes mit pandemiebedingten Restriktionen (Phase 2 [1, 8]; 16. März 2020–17. April 2020) wurde exakt der Vorjahreszeitraum (16. März 2019–17. April 2019) herangezogen. Referenzzeitpunkt ist das Aufnahmedatum. Es erfolgte eine deskriptive Analyse der Leistungszahlen der Klinik sowie eine Bewertung von Belegtagen mit den gesetzlich zugesicherten Kompensationszahlungen im Jahr 2020. Hierbei wird neben den gängigen Größen der Fallzahl, Casemix (CM) und Casemix-Index (CMI) auch der sog. Daymix-Index (DMI) berücksichtigt, also der CM eines Behandlungsfalls dividiert durch die Belegungstage. Weiterhin werden die Erlöse für die stationären Behandlungsfälle dargestellt und verglichen, da aufgrund der Ausgliederung der Pflegekosten ab 2020 ein direkter Vergleich von Relativgewichten alleine nicht zielführend wäre. Patienten im Jahr 2020, die noch stationär behandelt werden, werden mit ihrer aktuellen DRG bewertet. Als Verweildauer wird bei diesen Patienten die mittlere Verweildauer anhand des Fallpauschalenkatalogs 2020 angenommen. Begleitpersonen werden bei der Berechnung der Leistungszahlen nicht berücksichtigt. Privat- und BG-Patienten werden nach DRG abgerechnet und sind mitberücksichtigt. Zur Berechnung des Pflegeentgelts erfolgt die individuelle Multiplikation von Belegungstagen, Pflegerelativgewicht und Pflegeentgeltwert. Letzter beträgt bis zum 31. März 2020 146,55 € und ab 1. April 2020 185,00 €. Dies wurde bei den Monatsüberliegern 2020 entsprechend berücksichtigt.

Der Landesbasisfallwert (LBFW) beträgt für Sachsen 2019 3528,65 € und für 2020 3663,09 €. Zum Vergleich des DMI-Äquivalents, das aus der Kompensation ausbleibender Belegungstage resultiert, wird der DMI vor dem Beobachtungszeitraum 2020 herangezogen, wobei Jahresüberlieger von 2019 nach 2020 nicht betrachtet werden. Zur Sachkostenberechnung nach DRG wurde auf die LBFW-adjustierten Sachkosten je DRG 2020 zurückgegriffen.

## Ergebnisse

**Figure 1:**
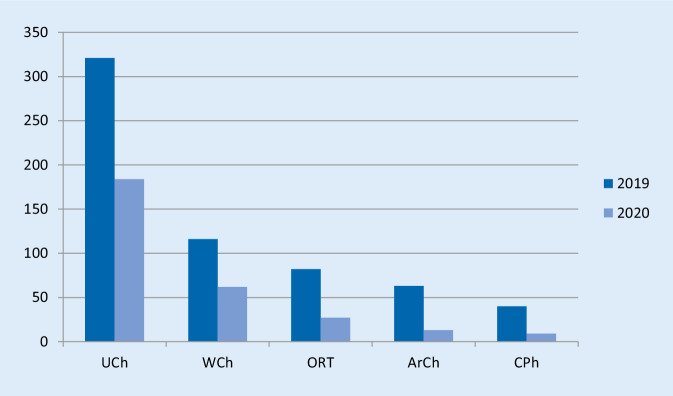

Im Untersuchungszeitraum 2019 wurden 622 Personen an der KOUP aufgenommen ([596 Patienten, 26 Begleitpersonen] 321 UCh, 116 WCh, 82 ORT, 63 ArCh, 40 CPh). Im gleichen Zeitraum 2020 wurden 295 Patienten aufgenommen ([289 Patienten, 6 Begleitpersonen] 184 UCh, 62 WCh, 27 ORT, 13 ArCh, 9 CPh). Die Abb. 1. zeigt die Relationen beider Zeiträume. Das bedeutet einen Rückgang der Fallzahl von insgesamt 53 % (UCh: 43 %, WCh: 47 %, ORT: 67 %, ArCh: 79 %, PCh: 78 %).

**Figure 2:**
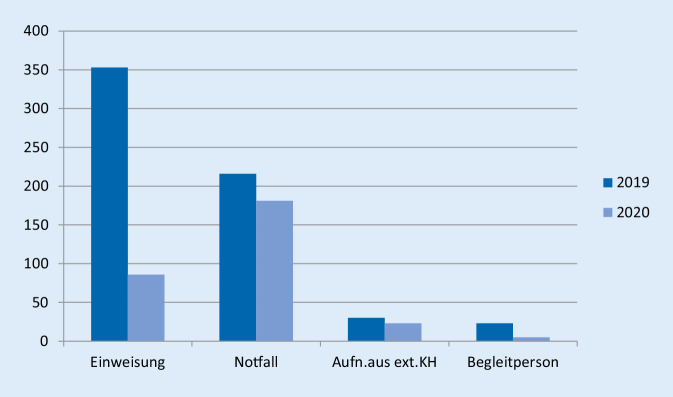

Die Differenzierung nach der Aufnahmeart ist in Abb. 2 und Tab. 1 dargestellt. Demnach lässt sich ein Rückgang der elektiven Einweisungen um 267 Fälle (76 %) feststellen. Notfälle gingen um 16 % zurück und auch bei Verlegungen aus externen Krankenhäusern (23 %) sowie Begleitpersonen (78 %) war ein Rückgang zu verzeichnen.

**Table 1:** 

Bereiche	2019	2020	∆ %
*UCh*	*321*	*184*	*−43*
Notfall	196	142	−28
Einweisung	78	32	−59
Begleitperson	23	4	−83
Zuweisung externes KH	24	6	−75
*WCh*	*116*	*62*	*−47*
Notfall	16	29	81
Einweisung	95	21	−78
Begleitperson	0	1	–
Zuweisung externes KH	5	11	120
*ORT*	*82*	*27*	*−67*
Notfall	1	2	100
Einweisung	79	23	−71
Begleitperson	1	1	0
Zuweisung externes KH	1	1	0
*ArCh*	*63*	*13*	*−79*
Notfall	1	5	400
Einweisung	60	6	−90
Begleitperson	2	0	−100
Zuweisung externes KH	0	2	–
*PCh*	*40*	*9*	*−78*
Notfall	2	3	50
Einweisung	38	3	−92
Zuweisung externes KH	0	3	–
*Aufnahmen gesamt*	*622*	*295*	*−53*

*ArCh* Arthroskopie/gelenkerhaltende Chirurgie, *KH* Krankenhaus, *ORT* Orthopädie, *PCh* Plastische Chirurgie, *UCh* Unfallchirurgie, *WCh* Wirbelsäulenchirurgie

Die 596 Behandlungsfälle 2019 (ohne Begleitpersonen) generierten 4602 Behandlungstage bei einem CM von 937 Punkten, einem CMI von 1,572 und einem DMI von 0,269. Daraus resultiert auf Basis des Landesbasisfallwerts 2019 ein Erlös von 3.305.890 € (Tab. 2). Demgegenüber resultieren bei den im Zeitraum 2020 behandelten Patienten 2004 Behandlungstage und ein CM von 342 mit zusätzlich 246 Pflegerelativgewichten. Es ergibt sich für 2020 ein CMI von 1,182 und ein DMI (ohne Pflege) von 0,171. Der erwartete Erlös für den Beobachtungszeitraum 2020 beträgt 1.434.820 €, was einem Rückgang gegenüber dem Vorjahr von 1.871.069 € bzw. 57 % entspricht. Dabei ist der erhöhte Pflegeentgeltwert ab 1. April 2020 für den Beobachtungszeitraum 2020 bereits berücksichtigt und beträgt in Summe 21.454 €.

**Table 2:** 

Bereich KOUP	Erlöse 2019	Erlöse 2020	∆
UCh	1.646.810	770.223	−876.588
WCh	624.151	400.176	−223.975
ORT	623.767	168.329	−455.438
ArCh	217.891	54.410	−163.481
CPh	193.271	41.683	−151.588
*Gesamtergebnis*	*3.305.890*	*1.434.820*	*−1.871.069*

*ArCh* Arthroskopie/gelenkerhaltende Chirurgie, *KOUP* Klinik für Orthopädie, Unfallchirurgie und Plastische Chirurgie, *ORT* Orthopädie, *PCh* Plastische Chirurgie, *UCh* Unfallchirurgie, *WCh* Wirbelsäulenchirurgie

Die Kompensation der nicht erbrachten Belegungstage der KOUP im Jahr 2020 gegenüber dem Vorjahr erfolgt durch 560 € pro Tag. Im Beobachtungszeitraum 2020 waren 2320 Belegungstage weniger als im Vorjahreszeitraum zu verzeichnen. Daraus ergibt sich eine Ausgleichsvergütung von 1.299.200 €. Aus diesen Betrachtungen resultiert unter Berücksichtigung monetär teilweise kompensierter Leerstände sowie des erhöhten Pflegeentgeltwertes ein Erlösrückgang von 572.071 €.

Zur Analyse der Ursache hierfür bietet sich der Vergleich von DMI und der tagesbezogenen Kompensationspauschale von 560 € an. Unter Berücksichtigung des LBFW 2020 ergibt sich aus 560 € pro Tag ein DMI von 0,153. Pflegeanteile werden hierbei nicht berücksichtigt, sodass zum Vergleich der DMI nach Bereich vor dem Beobachtungszeitraum 2020 heranzuziehen ist (Tab. 3).

**Table 3:** 

Bereich KOUP	DMI	DMI bei 560 € Tagespauschale	Unterdeckung DMI durch Tageschpauschale	Unterdeckung absolut je Fall
UCh	0,161	0,153	0,008	215,54 €
WCh	0,160	0,153	0,007	300,52 €
ORT	0,196	0,153	0,043	1269,94 €
ArCh	0,234	0,153	0,081	1313,20 €
CPh	0,265	0,153	0,112	1825,21 €
*Gesamtergebnis*	*0,179*	*0,153*	*0,026*	*703,74* *€*

*ArCh* Arthroskopie/gelenkerhaltende Chirurgie, *DMI* Daymix-Index, *KOUP* Klinik für Orthopädie, Unfallchirurgie und Plastische Chirurgie, *ORT* Orthopädie, *PCh* Plastische Chirurgie, *UCh* Unfallchirurgie, *WCh* Wirbelsäulenchirurgie

Der verhältnismäßig niedrige DMI für die WCh resultiert aus der Gesamtbetrachtung des Bereichs, also der konservativen und der operativen Wirbelsäulenchirurgie. Letztere alleine hat einen DMI von 0,213 gegenüber der konservativen WCh mit einem DMI von 0,100.

Es zeigt sich, dass die größten Unterschiede beim DMI die Bereiche Orthopädie, Arthroskopische Chirurgie und Plastische Chirurgie betreffen. Die daraus resultierende Unterdeckung je Fall beträgt für die Orthopädie 1269,94 €, für die Arthroskopische Chirurgie 1313,20 € und für die Plastische Chirurgie 1825,21 €.

Demnach müsste die tagesbezogene Kompensationspauschale etwa 656 € betragen, um einen DMI von 0,179 zu generieren. Damit wäre das Erlösniveau vor pandemiebedingten Restriktionen ausgeglichen. Für alle voll- und teilstationären Fälle können die Krankenhäuser ab 1. April 2020 zusätzlich 50 € abrechnen. Im Beobachtungszeitraum wurden 158 Patienten der KOUP seit dem 1. April aufgenommen. Daraus resultiert ein zusätzlicher Erlösposten in Höhe von 7900 €.

**Figure 3:**
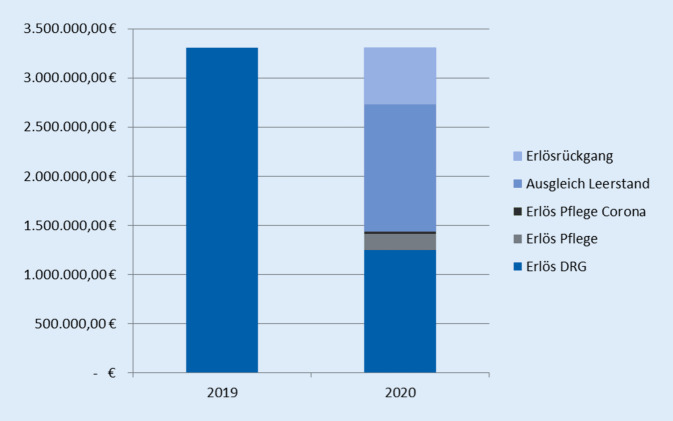

Abb. 3 stellt die einzelnen Komponenten der monetären Ausgleichsmechanismen dar, wobei die 50-€-Pauschale aufgrund ihrer geringen Gesamtsumme nicht darstellbar ist.

Nun sind aber noch die variablen Kosten zu betrachten, die für nichtaufgenommene Patienten nicht anfallen und somit auch nicht ausgeglichen werden müssten. Dazu wurde der Rückgang der Fallzahlen jeder einzelnen DRG des Beobachtungszeitraumes 2020 versus 2019 betrachtet und mit den variablen Kosten der Kostenarten 4a, 4b und 5 (Medikamente und Implantate) multipliziert. Daraus ergeben sich nicht angefallene variable Kosten in Höhe von 190.002 €. Diese sind von dem Erlösrückgang in Abzug zu bringen. Somit ergibt sich eine fehlende Kompensation zum Vorjahreszeitraum in Höhe von 382.069 €. Die Bereinigung um nicht angefallene Sachkosten ergibt als Erweiterung der Tab. 3 folgende Übersicht (Tab. 4):

**Table 4:** 

Bereich KOUP	DMI	DMI bei 560 € Tagespauschale	Unterdeckung DMI durch Tageschpauschale	Unterdeckung absolut je Fall	Summe Sachkosten	Unterdeckung bereinigt um Sachkosten
UCh	0,161	0,153	0,008	215,54 €	71.205,05 €	86,31 €
WCh	0,160	0,153	0,007	300,52 €	24.160,09 €	220,52 €
ORT	0,196	0,153	0,043	1269,94 €	80.092,90 €	888,55 €
ArCh	0,234	0,153	0,081	1313,20 €	11.349,63 €	1254,70 €
CPh	0,265	0,153	0,112	1825,21 €	3194,01 €	1801,89 €
*Gesamtergebnis*	*0,179*	*0,153*	*0,026*	*703,74* *€*	*190.001,69* *€*	*567,44* *€*

*ArCh* Arthroskopie/gelenkerhaltende Chirurgie, *DMI* Daymix-Index, *KOUP* Klinik für Orthopädie, Unfallchirurgie und Plastische Chirurgie, *ORT* Orthopädie, *PCh* Plastische Chirurgie, *UCh* Unfallchirurgie, *WCh* Wirbelsäulenchirurgie

Da in der vorliegenden Untersuchung ein 5‑Wochen-Zeitraum betrachtet wurde, ist eine Hochrechnung auch auf längere Zeiträume einfach: Der nichtkompensierte Erlösausfall beträgt pro Woche 76.414 € und somit pro Jahr 3.973.518 €.

## Diskussion

Die Corona-Pandemie stellt für das Gesundheitswesen eine enorme medizinische und wirtschaftliche Herausforderung dar [11]. Die Bundes- und Landespolitik unterstützt mit einem großen Maßnahmenkatalog, kann dabei aber allenfalls auf geringe Empirie zurückgreifen [4]. Zudem kommen der zeitliche und der öffentliche Druck hinzu, mit dem Entscheidungen von der Politik erwartet wurden. In diesem Zuge trat am 28. März das COVID-19-Krankenhausentlastungsgesetz in Kraft [4]. Darin werden zahlreiche monetäre Kompensationsmechanismen für Krankenhäuser geregelt, die meist rückwirkend Geltung haben. Diese Arbeit vergleicht den Zeitraum seit Beginn der restriktiven Maßnahmen am Uniklinikum Leipzig (Aussetzen elektiver Operationen, Reduktion stationärer Kapazitäten u. a.) mit dem gleichen Zeitraum des Vorjahres. Dieser bot keine Auffälligkeiten in den klinischen Leistungszahlen, sodass er als Referenz herangezogen werden kann. Die Wahl des Zeitraumes 2020 beruht auf der Maßgabe eines Beschlusses, der von der Bundeskanzlerin mit den Ministerpräsidenten der Länder am 12. März 2020 getroffen wurde [9]. Darin heißt es, dass „grundsätzlich alle planbaren Aufnahmen, Operationen und Eingriffe in allen Krankenhäusern ab Montag auf unbestimmte Zeit verschoben und ausgesetzt werden“ [9].

Dies entspricht den Vorgaben der AAOS und der DGU/DGOU entsprechend der Pandemiephase 2 [1, 8]. Auch an der KOUP wurde dieser Beschluss ab Montag, den 16. März 2020 umgesetzt. Hiernach kam es zu einem Rückgang der Aufnahmen von 53 % im Vergleich zum entsprechenden Vorjahreszeitraum. Dabei ist der Rückgang der stationären Notfallpatienten erwartungsgemäß geringer, als der Rückgang der (teil-)elektiven Einweisungen. Der prozentuale Fallzahlrückgang lässt sich differenzieren in Bereiche mit erfahrungsgemäß höherem Anteil an elektiven Patienten (ORT, ArCh, PCh) und hier entsprechend höherem Rückgang an Patientenaufnahmen, sowie in Bereiche mit höherem Anteil an dringlichen und Notfallindikationen zur Aufnahme (UCh, WCh).

Der Rückgang der unfallchirurgischen Notfälle lässt sich am ehesten auf die in Sachsen umgesetzte „Verordnung des Sächsischen Staatsministeriums für Soziales und Gesellschaftlichen Zusammenhalt zum Schutz vor dem Coronavirus SARS-CoV‑2 und COVID-19 (Sächsische Corona-Schutz-Verordnung – SächsCoronaSchVO) vom 31. März 2020“ zurückführen [12]. In diesem Zeitraum war jedweder physisch-soziale Kontakt zu anderen Menschen außerhalb der Angehörigen des eigenen Hausstands auf ein absolut nötiges Minimum zu reduzieren. Wo immer es möglich war, war ein Mindestabstand zwischen zwei Personen von mindestens 1,5 m einzuhalten [12]. Dies bedeutet, dass es insgesamt weniger Verkehr gab und damit weniger Verkehrsunfälle und durch das Kontaktverbot weniger Risikosportarten durchgeführt wurden. Durch die Einschränkungen des Berufslebens war auch der Anteil an berufsgenossenschaftlichen Unfällen regredient.

Insgesamt bedeuten die Restriktionen für die KOUP einen Erlösrückgang für stationäre Leistungen um 57 %. Durch die aktuelle Gesetzgebung wird hier gegengesteuert. Dabei trägt der erhöhte Pflegeentgeltwert nur einen marginalen Beitrag dazu bei. Das Gros des Erlösrückganges soll durch eine Pauschale von 560 € für jedes Bett, das im Zeitraum vom 16. März bis zum 30. September 2020 nicht belegt wird, ausgeglichen werden [5]. Der Ausgleich soll aus der Liquiditätsreserve des Gesundheitsfonds bezahlt werden [5, 6]. Dabei erfolgt keine Differenzierung nach Krankenhausgröße, Hauptabteilung oder einem anderen Schlüssel [5, 6]. In die Vergleichsgröße DMI umgerechnet zeigt sich, dass kein Bereich der KOUP eine auskömmliche Kompensation durch die 560 € Tagespauschale erhält. Die durchschnittliche Unterdeckung beträgt auch mit der Tagespauschale und trotz Berücksichtigung einer Sachkostenkorrektur 567 € je Fall.

Weiterhin stellt die Aufwertung des Pflegeentgeltwertes auf 185 € für den Beobachtungszeitraum keine ausreichende Kompensation dar. Grundlegend sind die Pflegeerlöse als Abschlagszahlung auf das Pflegebudget zu verstehen. Das bedeutet, dass alle Pflegeerlöse zwar akut liquiditätswirksam sind, jedoch letztendlich mit dem testierten und verhandelten Pflegebudget abgeglichen werden.

Weitere Kompensationsmaßnahmen des COVID-19-Krankenhausentlastungsgesetzes, wie zum Beispiel das Aussetzen des Fixkostendegressionsabschlags für 2020, sind zwar hilfreich [6], auf einen einzelnen Bereich und kurzen Zeitraum aber nicht ohne weiteres geldwert umzurechnen. Die in §21 Abs. 6 KHG geregelte Corona-Mehrkostenpauschale von 50 € pro Patient fällt bei der vorliegenden Betrachtung kaum ins Gewicht [6]. Außerdem sind Erlösausfälle in den Hoschschulambulanzen überhaupt nicht adressiert, ein Umstand, der sich bei der bereits vor der Pandemie bestehenden Unterdeckung nun weiter verschlechtert [10]. Erfreulich ist allerdings die Verpflichtung der Kostenträger durch den Gesetzgeber zu einer verkürzten Zahlungsfrist von 5 Tagen nach Zugang der Krankenhausrechnung. Jedoch unterstützt auch dieser Schritt lediglich die Liquidität der Krankenhäuser und stellt kein zusätzliches Geld zur Verfügung [7]. Auch die verringerte Prüfquote für den Medizinischen Dienst (MD) und das Aussetzen der Sanktionszahlungen für beanstandete Fälle durch den MD ist zu begrüßen [4].

Zusammenfassend ist festzuhalten, dass es dem Gesetzgeber gelungen ist, sichtbare wirtschaftliche Unterstützung für die Krankenhäuser zu gewähren [6]. Die aufgeführten Mechanismen reichen aber keineswegs, um Erlösrückgänge von Kliniken für Orthopädie und Unfallchirurgie zu kompensieren. Das liegt aus Sicht der Autoren maßgeblich an der undifferenzierten Ausgestaltung der Kompensationszahlung für Leerbetten. Hier wäre zumindest für die Leerbettenkompensation eine Orientierung am DMI für verschiedene Hauptabteilungen sowie eine Berücksichtigung der Versorgungsstufe oder Bettenzahl wünschenswert. Weiterhin ist die nahezu vollständige Einstellung des elektiven Ambulanzgeschehens und daraus resultierend auch die Reduktion zumindest kurz- und mittelfristiger Einweisungen äußerst bedrohlich [8, 12].

Ein wenig Hoffnung gibt das in Aussicht gestellte „Zweite Gesetz zum Schutz der Bevölkerung bei einer epidemischen Lage von nationaler Tragweite“. Hier soll u. a. das Prüfgeschehen durch die Kostenträger bis Ende 2022 weiterhin restriktiv geregelt werden. Zudem ist eine Aufstockung der Leerbettenkompensation durch die Länder im Gespräch [2].

## Limitationen

Eine Bewertung zur Auskömmlichkeit der verschiedenen Kompensationsmechanismen ist nach dem relativ kurzen Zeitraum abschließend nicht möglich. Außerdem werden in der vorliegenden Arbeit nur stationäre Fälle betrachtet. Insbesondere eine teilweise verstärkte Wiederaufnahme des Operationsgeschehens einzelner Bereiche müsste eine erneute Bewertung nach sich ziehen.

Die Aussage für ein gesamtes Klinikum ist aus der Betrachtung einzelner Bereiche nicht möglich.

## Fazit für die Praxis

- Die Bundesregierung reagiert mit einem Maßnahmenpaket zur wirtschaftlichen Unterstützung der Krankenhäuser auf die Corona-Pandemie.
- Die Kompensationsmechanismen haben unterschiedliche Ansatzpunkte.
- Orthopädie und Unfallchirurgie sind aufgrund der vorgeschriebenen Verschiebung elektiver Behandlungen stark von der Pandemie betroffen.
- Die wirtschaftliche Unterstützung durch das COVID-19-Krankenhausentlastungsgesetz ist für Orthopädie und Unfallchirurgie nicht auskömmlich.

## Abkürzungen

AAOS: American Academy of Orthopaedic Surgeons
ArCh: Arthroskopie/gelenkerhaltende Chirurgie
BG: Berufsgenossenschaft
CM: Casemix
CMI: Casemix-Index
COVID 19: Corona Virus Disease 2019
DGOU: Deutsche Gesellschaft für Orthopädie und Unfallchirurgie
DGU: Deutsche Gesellschaft für Unfallchirugie
DMI: Daymix-Index
DRG: Diagnosis Related Groups
KH: Krankenhaus
KOUP: Klinik für Orthopädie, Unfallchirurgie und Plastische Chirurgie
LBFW: Landesbasisfallwert
MD: Medizinischer Dienst
ORT: Orthopädie
PCh: Plastische Chirurgie
UCh: Unfallchirurgie
WCh: Wirbelsäulenchirurgie
